# Drug Transport at the Brain and Endothelial Dysfunction in Preeclampsia: Implications and Perspectives

**DOI:** 10.3389/fphys.2018.01502

**Published:** 2018-11-06

**Authors:** Pablo Torres-Vergara, Carlos Escudero, Jeffrey Penny

**Affiliations:** ^1^Department of Pharmacy, Faculty of Pharmacy, University of Concepción, Concepción, Chile; ^2^Group of Research and Innovation in Vascular Health (GRIVAS Health), Chillán, Chile; ^3^Vascular Physiology Laboratory, Department of Basic Sciences, Faculty of Basic Sciences, Universidad del Bío-Bío, Chillán, Chile; ^4^Red Iberoamericana de Alteraciones Vasculares Asociadas a Trastornos del Embarazo (RIVA-TREM), Chillán, Chile; ^5^Division of Pharmacy and Optometry, School of Health Sciences, Faculty of Biology, Medicine and Health, The University of Manchester, Manchester, United Kingdom

**Keywords:** blood-brain barrier, ABC transporters, SLC transporters, tight junction proteins, endothelial dysfunction, preeclampsia, eclampsia, brain alterations

## Abstract

Transport of drugs across biological barriers has been a subject of study for decades. The discovery and characterization of proteins that confer the barrier properties of endothelia and epithelia, including tight junction proteins and membrane transporters belonging to the ATP-binding cassette (ABC) and Solute Carrier (SLC) families, represented a significant step forward into understanding the mechanisms that govern drug disposition. Subsequently, numerous studies, including both pre-clinical approaches and clinical investigations, have been carried out to determine the influence of physiological and pathological states on drug disposition. Importantly, there has been increasing interest in gaining a better understanding of drug disposition during pregnancy, since epidemiological and clinical studies have demonstrated that the use of medications by pregnant women is significant and this condition embodies a series of significant anatomical and physiological modifications, particularly at excretory organs and barrier sites (e.g., placenta, breast) expressing transporter proteins which influence pharmacokinetics. Currently, most of the research in this field has focused on the expression profiling of transporter proteins in trophoblasts and endothelial cells of the placenta, regulation of drug-resistance mechanisms in disease states and pharmacokinetic studies. However, little attention has been placed on the influence that the cerebrovascular dysfunction present in pregnancy-related disorders, such as preeclampsia, might exert on drug disposition in the mother’s brain. This issue is particularly important since recent findings have demonstrated that preeclamptic women suffer from long-term alterations in the integrity of the blood-brain barrier (BBB). In this review we aim to analyze the available evidence regarding the influence of pregnancy on the expression of transporters and TJ proteins in brain endothelial cells, as well the mechanisms that govern the pathophysiological alterations in the BBB of women who experience preeclampsia. Future research efforts should be focused not only on achieving a better understanding of the influence of preeclampsia-associated endothelial dysfunction on drug disposition, but also in optimizing the pharmacological treatments of women suffering pregnancy-related disorders, its comorbidities and to develop new therapies aiming to restore the integrity of the BBB.

## Introduction

Worldwide, there has been an increase in the number of prescribed and over-the-counter medications taken by pregnant women (Mitchell et al., 2011; Beyene and Beza, 2018; Navaro et al., 2018). In Latin America, the true extent of the use of therapeutic drugs among pregnant women is not well characterized, but in countries such as Uruguay, 96% of pregnant women take medications and 78% use two or more (Viroga et al., 2013). A prospective cohort study conducted in Brazilian, Argentinian and Peruvian populations showed that immunodeficiency virus (HIV)-infected pregnant women exhibit a better adherence to anti-HIV therapy when compared to post-partum (Kreitchmann et al., 2012). The above-mentioned statistics are significant since pregnancy is a physiological condition associated with anatomical and physiological modifications capable of influencing the disposition of drugs, including increased blood volume, enhanced basal metabolism, and modified hormone levels, among others.

Treatment of chronic diseases within pregnancy carries a risk for both the mother and fetus, as the administered drug could cross the placenta and reach the fetus circulation, with deleterious consequences (Jentink et al., 2010; Tomson et al., 2018). Pharmacokinetic studies carried out in animal models and human suggest that exposure to drugs is reduced in pregnancy since there is an increase in both the renal glomerular filtration rate (thereby increasing renal elimination) and hepatic metabolism mediated by isoforms of cytochrome P450 enzymes and uridine 5′-phosphate glucuronosyltransferases (Pariente et al., 2016; Koren and Pariente, 2018). Furthermore, the expression and activity of transporters involved in drug disposition appears to be modified in excretory organs (Hebert et al., 2008) in a similar fashion to that observed with metabolizing enzymes.

Pregnant women suffering from psychiatric disorders and other central nervous system (CNS) diseases often require pharmacotherapy to stabilize their symptoms, which are likely to continue after labor due to their chronic nature. Clinical studies of pregnant women receiving antiepileptic and antidepressive pharmacotherapy demonstrated that the reduced drug exposure, due to increased clearance, is associated with an increase in the seizure rate (Reisinger et al., 2013) and decreased plasma levels of serotonin reuptake inhibitors, respectively (Westin et al., 2017). These outcomes clearly demonstrate that pregnancy could have a negative impact on the clinical effect of these drugs.

Furthermore, women under pharmacological treatment for epilepsy (Borthen, 2015) or depression (Palmsten et al., 2012) have a higher risk of suffering complications derived from pathophysiological alterations associated with pregnancy, such as preeclampsia. This pathological condition, that is present in 2–8% of all pregnancies (Duley, 2009), is a disorder characterized by hypertension and proteinuria after the twentieth gestational week, which may evolve to vasogenic edema, eclampsia (seizures) and cerebrovascular stroke if not properly controlled (American College of Obstetricians and Gynecologists, Task Force on Hypertension in Pregnancy, 2013; Cipolla, 2013; Hammer and Cipolla, 2015). It is also reported that 75% of maternal deaths due to preeclampsia are related to cerebrovascular complications including eclampsia, intracranial hemorrhage, and edema (Zeeman, 2009).

Preeclampsia is associated with impaired systemic endothelial function (Roberts et al., 1989) and, in the brain of preeclamptic women, this endothelial dysfunction presents in the form of impaired integrity of the BBB (Bergman et al., 2018), which is apparently maintained even post-partum (Bergman et al., 2016). The influence of endothelial dysfunction on drug disposition in the brain has been studied in disease states, including stroke (Huang et al., 2017) however there is a lack of studies investigating the effect of endothelial dysfunction on brain drug disposition in pregnancy-related disorders. The latter issue is extremely important and needs to be addressed since preeclamptic women may need to receive medications to control symptoms within pregnancy, and/or at some point post-partum, particularly for treatment of chronic conditions, e.g., epilepsy, depression and HIV-infection. Furthermore, it is likely that women with brain endothelial dysfunction could experience increased exposure to the effects of endogenous factors and potentially harmful xenobiotics.

While the function of the BBB and transport of molecules across brain endothelial cells (BECs) has been extensively studied in non-pregnant populations, much less is known about BBB physiology during pregnancy and pregnancy-related disorders. Therefore, this review will summarize the findings related to the effect of pregnancy and preeclampsia on the expression and activity of proteins involved in the transport of drugs at the BBB.

## Overview of the Blood-Brain Barrier

**FIGURE 1:**
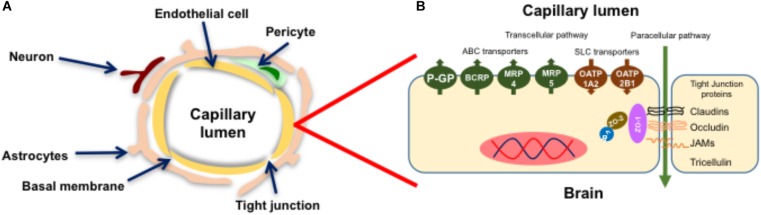
Structure of the blood-brain barrier. The BBB is a neurovascular unit comprised of endothelial cells, a basal membrane, pericytes, astrocytes and neurons **(A)**. Brain endothelial cells (BECs) express ABC/SLC transporters and high levels of Tight Junction (TJ) proteins that confer the barrier properties of the BBB **(B)**. Figure adapted from Abbott et al. (2010) and O’Brien et al. (2012) with permission from their publishers.

The BBB (Figure 1A) is a highly restrictive and specialized neurovascular network comprised of BECs, a basal membrane composed on collagen, fibronectin and laminin, pericytes, neurons and glial cells (Abbott, 2013; Daneman and Prat, 2015). In essence, the BBB isolates the brain parenchyma from the systemic circulation, regulating the supply of nutrients, controlling the bidirectional transport of endogenous mediators and protecting the CNS from exposure to harmful compounds, i.e., xenobiotics and metabolites.

Unlike other vascular beds, the endothelial cells of the BBB express a unique phenotype (Figure 1B) with higher levels of expression of tight junction (TJ) proteins, membrane transporters belonging to the ATP-binding cassette (ABC) and Solute Carrier families (SLC), and metabolizing enzymes (Decleves et al., 2011; Shawahna et al., 2011; Daneman and Prat, 2015; Liao et al., 2017).

Findings of *in vitro* and animal models have consistently demonstrated that the transport of molecules across the BBB is strongly regulated by membrane transporters and TJ proteins (Cecchelli et al., 1999; Cantrill et al., 2012; Helms et al., 2016). More recently, the use of imaging techniques and probes have allowed the *in vivo* analysis of transporter functionality, e.g., P-glycoprotein (P-GP) activity at the BBB in both healthy (Bauer et al., 2015) and disease states (Shin et al., 2016).

## Overview of Tight Junction Proteins in Brain Endothelial Cells

The paracellular transport of molecules at the BBB is highly selective, and this feature is associated with the expression of high levels of TJ proteins. These proteins act as a biological adhesive, anchoring together adjacent BECs via transmembrane proteins attached to intracellular scaffolding proteins (Haseloff et al., 2015).

The transmembrane proteins occludin, claudins and Junctional Adhesion Molecules (JAMs) form complex strands that interact between cells, reducing paracellular diffusion (Haseloff et al., 2015; Keaney and Campbell, 2015). However, in order to maintain this restrictiveness, TJs are linked to the cytoplasmic zonula occludens (ZO) proteins that provide a structural bridge to the actin cytoskeleton. In zones where there is contact between three BECs, the TJ protein tricellulin (MARVELD2) plays a pivotal role in modulating paracellular permeability by reducing the passage of large molecules (Reinhold and Rittner, 2017).

Since the discovery that the permeability of the BBB could be regulated through reversible disruption of TJs, this principle has served as an approach for the delivery of therapeutics that would not cross this barrier by conventional means, e.g., passive diffusion, carrier-mediated transport (Dithmer et al., 2017; Sol et al., 2017).

## Overview of Transporters Involved in Brain Drug Disposition

Data from *in vitro* and animal models have helped establish which membrane transporters impact brain drug disposition. Furthermore, the International Transporter Consortium et al. (2010) has published and updated recommendations (Hillgren et al., 2013) for decision-making processes related to drug-transporter interactions that could be translated to clinical settings. In this regard, the ABC transporters P-glycoprotein (P-GP; Cordon-Cardo et al., 1989), Multidrug Resistance-associated Proteins (MRPs) MRP4 and MRP5 (Huai-Yun et al., 1998; Seetharaman et al., 1998), Breast Cancer Resistance Protein (BCRP; Eisenblatter and Galla, 2002; Eisenblatter et al., 2003), and the Organic Anion Transporting Polypeptides (OATPs) OATP1A2 and OATP2B1 SLC transporters (Roth et al., 2012), are considered the most clinically important transporters within the BBB. Although there is evidence (from pre-clinical models) that other SLC transporters expressed in the BBB, including members of the Monocarboxylate Transporter (MCT; Lee and Kang, 2016), Organic Anion Transporter (OAT) subfamilies (Hosoya and Tachikawa, 2011) are involved in the uptake of drugs, this review will exclusively focus on the transporter proteins expressed in human BECs. The characteristics of these protein families in human BECs are briefly summarized in Table 1 and the following section.

**Table 1:** Human BEC ABC and SLC transporters involved in drug disposition.

Transporter	Gene	Molecular weight (kDa)	Detected at protein level	Localization and function	References
**ABC**					
P-GP	ABCB1	170	Yes	Luminal/Efflux	Dauchy et al., 2009; Shawahna et al., 2011; Uchida et al., 2011
					
BCRP	ABCG2	70 (Monomer)	Yes	Luminal/Efflux	Eisenblatter et al., 2003; Dauchy et al., 2009; Shawahna et al., 2011; Uchida et al., 2011
					
MRPs	ABCC4	170	Yes	Luminal/Efflux	Nies et al., 2004; Shawahna et al., 2011
	ABCC5	160	Yes		
					
**SLC**					
OATP1A2	SLC21A3	≈70	Yes	Luminal/Uptake	Lee et al., 2005; Roth et al., 2012
					
OATP2B1	SLC21A9	≈77	Yes	Luminal/Uptake	Bronger et al., 2005; Roth et al., 2012

### ATP-Binding Cassette Transporters

P-glycoprotein is a 170 kDa efflux transporter encoded by the *ABCB1* gene in human and the *abcb1a/abcb1b* genes in rodent. This protein is located at the luminal side of BECs and is described as a phenotypical marker (Sugawara et al., 1990; Dauchy et al., 2008; Cantrill et al., 2012). P-GP exhibits a broad substrate specificity that includes anticancer drugs (Mealey and Fidel, 2015), antidepressant drugs (O’Brien et al., 2012), antiepileptic drugs (Stepien et al., 2012), cardiotonic drugs (Ledwitch et al., 2016), HIV protease inhibitors (Liu et al., 2017) and immunosuppressants (Picchianti-Diamanti et al., 2014) among others. This characteristic implies the transporter has a predominant role in regulation the disposition of xenobiotics, including therapeutic drugs, thereby acting as a mechanism of detoxification and drug resistance. Some studies have also proposed a role for P-GP in the transport of endogenous mediators including steroids, bilirubin (Cascorbi, 2011) and amyloid-β, the peptide responsible of the formation of amyloid plaques in Alzheimer’s disease (Zhong et al., 2016).

The BCRP is an ABC transporter encoded by the *ABCG2* gene in human and *abcg2* gene in rodent, and is expressed at the luminal domain of BBB endothelial cells (Eisenblatter et al., 2003). BCRP is a monomeric protein (70 kDa) that requires the formation of at least a homodimer (and can even form homotetramers) to be functionally active (Ni et al., 2010). There is significant overlap in the substrate specificity of P-GP and BCRP and, like P-GP, BCRP can significantly influence drug transport in the body (Poguntke et al., 2010). In human BECs, BCRP is expressed at higher levels than P-GP (Shawahna et al., 2011; Uchida et al., 2011), but its overall contribution to the transport of substrates is less well understood than P-GP. As well as transporting drug substrates, BCRP also participates in transport of hormones (and conjugated metabolites) (Grube et al., 2018) and urate, a product of purine metabolism whose accumulation causes gout (Woodward et al., 2009; Fujita and Ichida, 2018).

The MRP transporters are encoded by the *ABCC* class of genes in human and *abcc* genes in rodents. MRPs mediate the transport of a diverse array of drugs and endogenous molecules including hormones, prostaglandins, leukotrienes and their conjugates (glucuronides, sulfates, and glutathione) (Zhou et al., 2008; Zhang et al., 2015; Bloise et al., 2016). Members of the MRP family are not as highly expressed in BECs as P-GP and BCRP in human BECs, but the findings of proteomic (Shawahna et al., 2011; Uchida et al., 2011) and transcriptomic (Warren et al., 2009) analyses have demonstrated that MRP4, an isoform located at the luminal side, is expressed at detectable levels in human BECs. Luminal expression of MRP5 in human BECs has also been confirmed by means of fluorescent immunohistochemistry (Nies et al., 2004).

### Solute Carrier Transporters

The OATPs, a group of SLC transporters belonging to the SLCO subfamily, are ubiquitously expressed throughout the body, and at the human BBB, luminal expression of OATP1A2 (Lee et al., 2005) and OATP2B1 (Bronger et al., 2005), has been reported. OATPs mediate the uptake and efflux of endogenous and exogenous molecules, which tend to possess amphiphilic characteristics (Roth et al., 2012) and substrates include prostaglandins, steroid and thyroid hormone conjugates (Grube et al., 2018), bile acids and therapeutic drugs (Kalliokoski and Niemi, 2009; Roth et al., 2012).

## Expression and Functionality of Blood-Brain Barrier Tight Junctions Proteins and Drug Transporters in Pregnancy

During normal pregnancy there is an increase in blood levels of several endogenous mediators including hormones and their metabolites, pro-inflammatory cytokines, chemokines, matrix metalloproteinases and growth factors (Chavan et al., 2017; Chen and Khalil, 2017). For example, clinical studies report that pro-inflammatory cytokines including interleukin-6(IL-6) and markers of cyclooxygenase-2 activity were increased in healthy women, suggesting that pregnancy is characterized by a mild, sub-clinical systemic inflammatory state (Palm et al., 2013; Danielsen et al., 2014). Under this physiological pro-inflammatory condition, expression and functionality of BBB TJ proteins and ABC/SLC transporters involved in drug disposition could be modified as is observed in other conditions (Keaney and Campbell, 2015; Qosa et al., 2015). Although little is known of the influence of pregnancy on the expression of BBB transporters and TJ proteins, recent studies are addressing this issue.

### Tight Junction Proteins and Pregnancy

The influence of pregnancy on the integrity of TJs has primarily been studied in endothelial cells of the placenta (Marzioni et al., 2001; Ahn et al., 2015), and to date, we are not aware of any studies reporting the effects of a healthy pregnancy on the expression of TJ proteins in the maternal BBB. During normal pregnancy, high plasma levels of vascular endothelial growth factor (VEGF), an angiogenic mediator that increases BBB permeability through changes in the expression of TJ proteins (Lafuente et al., 2006) including claudin-5 and occludin (Argaw et al., 2012), have been reported (Evans et al., 1998). However, despite elevated plasma levels of VEGF, the permeability of the BBB remains unaltered in normal pregnancy (Cipolla, 2013). Indeed, studies have shown that the serum collected during late pregnancy attenuates the effects of VEGF on permeability of cerebral veins isolated from non-pregnant rats and rats in late-pregnancy (Schreurs et al., 2012). The authors attributed this outcome to the fact that, in late pregnancy, high plasma levels of soluble Fms-like tyrosine kinase 1 (sFlt1), a splice variant of the VEGF receptor (VEGFR) lacking activity, counteracts the effects of VEGF (Cipolla, 2013).

### Membrane Transporters

Current evidence suggests that expression levels of ABC transporters within the maternal BBB vary throughout pregnancy, although this outcome has only been demonstrated in animal models. Studies report the expression of rodent P-gp and mrp1 in the BBB of pregnant mice is higher at mid-gestation and decreases in late-gestation (Coles et al., 2009), whilst positron emission tomography (PET) studies conducted on macaques (Chung et al., 2010) report that P-GP activity increased from mid-gestation to late-gestation, as evidenced by reduced accumulation of the radiolabelled P-GP substrate ^11^C-verapamil. However, the latter study did not confirm if this effect was a result of increased P-GP expression. No studies to date have reported the influence of pregnancy on the expression of SLC transporters in the maternal BBB, although it has been demonstrated expression of rodent Oatp1a4 (a rodent isoform that shows a high homology with human OATP1A2) in the BBB of the newborn increases with maturation (Harati et al., 2013).

ATP-binding cassette and SLC transporters govern movement of a whole array of endogenous and exogenous molecules through endothelial cells of the BBB. Consequently, this transcellular passage of substances may be significantly affected by pregnancy-dependent changes in transporter expression. However, despite the potential implications of modification of the barrier properties of the maternal BBB, to date, the precise mechanisms by which pregnancy could influence the expression of the above transporters are unclear and are only relatively recently being investigated.

A recent study reported that acute exposure of isolated hippocampal rat brain capillaries to serum obtained from pregnant rats reduced P-GP activity (Johnson et al., 2018). The authors hypothesized that this inhibition of P-GP, mediated by high levels of circulating serum factors, was associated with the increased incidence of seizures in normal pregnant rats. However, this study did not identify the molecules responsible for reduced P-GP activity. Furthermore, the findings are in contrast to those reported in studies investigating the relationship between seizures and P-GP activity, which suggest glutamate-mediated induction of cyclooxygenase-2 activity is responsible for the up-regulation of P-GP expression and activity in BECs in animal models of epilepsy (Zibell et al., 2009; van Vliet et al., 2010) and in capillaries isolated from human brains (Avemary et al., 2013). These findings are particularly important since studies suggest that in normal pregnancies and in preeclampsia, the cerebral levels of glutamate are reduced when compared to non-pregnant women (Nelander et al., 2018).

The specific effects of endogenous factors on the expression and activity of ABC transporters during pregnancy have been studied more in-depth on the developing fetal BBB than in the maternal BBB. Studies have reported that primary cultures of guinea pig BECs, obtained from late-gestational fetuses and postnatal pups, are highly responsive to the effects of glucocorticoids and pro-inflammatory cytokines, with hydrocortisone and dexamethasone (Iqbal et al., 2011) increasing P-gp activity and IL-1β, IL-6 and TNFα (Iqbal et al., 2012) decreasing P-gp activity. However, despite the opposing effects exerted by glucocorticoids and cytokines on P-gp expression and activity, a later report demonstrated that co-treatment with the synthetic glucocorticoid dexamethasone, apart from increasing the expression of the transporter, enhanced the inhibitory actions of IL-1β, IL-6, and TNFα on P-GP activity (Iqbal et al., 2016). The authors suggested that this enhancement of cytokine inhibitory actions is the result of a dexamethasone-mediated increase in the expression of pro-inflammatory cytokines receptors.

Transforming Growth Factor β (TGFβ), a protein found at high levels in plasma during pregnancy (Forbes and Westwood, 2010), has also been reported to regulate the expression of P-GP in BECs, and Baello et al. (2016), have reported TGFβ-mediated up-regulation of P-GP expression and activity, through activation of the ALK1 and ALK5 signaling pathways, in BECs isolated from male fetuses and postnatal guinea pig pups.

## Endothelial Dysfunction at the Brain in Pregnancy-Related Disorders

Pregnancy alone can be considered as an inflammatory (but not pathological) state. One hallmark of pregnancy-related disorders, including preeclampsia, is the manifestation of endothelial dysfunction promoted by high levels of factors released from the placenta (Escudero et al., 2009; Myatt and Roberts, 2015).

The brain vasogenic edema present in later stages of preeclampsia is apparently the result of impaired autoregulation of cerebral blood flow and increased BBB permeability (Cipolla and Kraig, 2011; Cipolla, 2013; Hammer and Cipolla, 2015), but the pathophysiological mechanisms involved are still unclear. *In vitro* studies have demonstrated that when rat cerebral vasculature was exposed to plasma from normal and preeclamptic human pregnancies, there was an increase in BBB permeability. Interestingly, this effect was more marked following treatment with preeclamptic plasma (Amburgey et al., 2010). Furthermore, this study demonstrated that inhibition of VEGF receptor tyrosine kinase activity reversed the effect elicited by the treatment with preeclamptic plasma, suggesting that VEGF could be involved in modulating vascular permeability.

The pro-inflammatory cytokine TNFα is also believed to contribute to the increased BBB permeability in preeclampsia. TNFα infusion in healthy pregnant rats at gestational day 19 increased the water content in the anterior cerebrum without increasing the BBB permeability (Warrington et al., 2015). However, when pregnant rats were subjected to a reduction of uterine perfusion pressure (RUPP), a model of placental ischemia that emulates preeclampsia and impairs the maternal cerebral blood flow, they exhibited an increase in both the water content at the anterior cerebrum and BBB permeability, which was counteracted by treatment with the TNFα inhibitor etanercept.

Findings from other studies conducted in the RUPP model have helped to elucidate how BECs respond to the circulating factors present in preeclampsia. When RUPP was performed in pregnant rats at gestational day 14, edema and increased maternal BBB permeability in the anterior cerebrum were observed, with increased expression of the protein aquaporin 4 and no changes in the expression of TJ proteins (Warrington et al., 2014). However, it has been reported the same procedure led to post-partum edema and increased maternal BBB permeability in the posterior cortex, probably due to reduced expression of the TJ protein occludin (Clayton et al., 2018).

The increased BBB permeability reported by both Warrington et al. (2014) and Clayton et al. (2018), is partly supported by clinical studies which demonstrated that in women developing preeclampsia, blood levels of S100B, neuronal specific enolase (NSE) and neurofilament light chain (NfL), three markers of cerebral injury, were higher than those observed in women with normal pregnancies (Bergman et al., 2018). Indeed, another report showed that in preeclamptic women, the levels of S100B and NSE were still high 1-year post-partum, suggesting that the alterations in the integrity of the BBB are manifest for a substantial period of time following delivery (Bergman et al., 2016).

## Pharmacological Management of Preeclampsia

Hypertension is one of the pathological features of preeclampsia and is routinely managed with the use of antihypertensive drugs including nifedipine, nicardipine, labetalol, hydralazine and methyldopa (Odigboegwu et al., 2018). Although it remains unclear whether the BBB permeability of these drugs is affected during preeclampsia, it is noteworthy that several antihypertensives, namely labetalol, nicardipine and nifedipine, are substrates of efflux transporters including P-GP (Thiel-Demby et al., 2009; Choi et al., 2013; Incecayir et al., 2013). The therapeutic management of neurological complications associated with preeclampsia, including seizures, primarily relies on the intravenous or intramuscular administration of magnesium sulfate, a drug with demonstrated ability to prevent the development of eclampsia and reduce maternal mortality (Altman et al., 2002).

The mechanism of action by which magnesium sulfate exerts its effects is unknown, but despite this limitation, it is widely considered a neuroprotective agent capable of reducing BBB permeability in animal models of brain injury (Li et al., 2017). Furthermore, a recent study demonstrated that magnesium sulfate reduced the water content in the anterior cerebrum, as well protein, cytokine, chemokine and VEGF levels in cerebrospinal fluid of rats subjected to the RUPP procedure (Zhang and Warrington, 2016). The above findings are important since the transport of drugs and endogenous mediators across the choroid plexus, which constitutes the blood-cerebrospinal fluid barrier, is a subject that is receiving increasing interest. A better characterization of the mechanisms that govern transport of molecules across this barrier will certainly help to understand brain drug disposition in both healthy and disease states such as preeclampsia.

To date, as the specific effects of magnesium sulfate on the expression/functionality of BBB TJ proteins and drug transporters are unknown, future studies addressing this subject will prove crucial in identifying potential therapeutic targets and in developing treatment strategies.

## How Blood-Brain Barrier Endothelial Dysfunction Could Alter Brain Drug Disposition in Preeclampsia

The effect of endothelial dysfunction elicited by preeclampsia on brain drug disposition is unknown. A preeclampsia-mediated increase in BBB permeability could potentially result in increased permeation of endogenous, blood-borne substances, including placental derived sFlt-1, and hormones, into the brain. Furthermore, a less restrictive BBB could allow more extensive penetration of therapeutic drugs into the CNS, resulting in increased side effects. In preeclamptic women, in addition to hypertension, which is treated with antihypertensive P-GP drug substrates, comorbidities, including epilepsy, depression and HIV infection, are often reported (Pariente et al., 2016). Since there is evidence that drugs belonging to these pharmacological groups are substrates and/or of ABC/SLC transporters (O’Brien et al., 2012; Stepien et al., 2012; Alam et al., 2016; Han et al., 2017), there is an obvious need to better understand the effect of preeclampsia on BBB transporter physiology and the effects of transporter modifications on brain drug disposition. A list of drugs used for treatment of chronic diseases in pregnancy and preeclampsia is presented in Table 2.

**Table 2:** Drugs employed for treatment of chronic diseases in pregnancy and preeclampsia as substrates/inhibitors of ABC/SLC transporters expressed in human brain endothelial cells.

Drug	ABC Transporter		SLC Transporter		Reference
	Substrate	Inhibitor	Substrate	Inhibitor	
**Antidepressants**					
Citalopram		P-GP			Weiss et al., 2003a; O’Brien et al., 2012
Fluoxetine		P-GP			Weiss et al., 2003a; O’Brien et al., 2013
Paroxetine	P-GP	P-GP			Weiss et al., 2003a; O’Brien et al., 2012
**Antiepileptics**					
Carbamazepine	P-GP	P-GP			Weiss et al., 2003b; Zhang et al., 2012
Lamotrigine	P-GP, BCRP	P-GP			Weiss et al., 2003b; Luna-Tortos et al., 2008; Romermann et al., 2015
Oxcarbazepine	P-GP				Weiss et al., 2003b; Zhang et al., 2011; Antunes Nde et al., 2016
Phenobarbital	P-GP				Luna-Tortos et al., 2008
**Antihypertensives**					
Labetalol	P-GP				Incecayir et al., 2013
Nicardipine	P-GP				Kadono et al., 2010
Nifedipine	P-GP				Choi et al., 2013
**Antirretroviral drugs**					
Abacavir	P-GP, BCRP	P-GP, BCRP			Pan et al., 2007; Storch et al., 2007
Atazanavir	P-GP	P-GP, BCRP		OATP2B1	Perloff et al., 2005; Storch et al., 2007; Weiss et al., 2007; Fujimoto et al., 2009
Darunavir	P-GP	P-GP	OATP1A2	OATP2B1	Annaert et al., 2010; Hartkoorn et al., 2010; Kis et al., 2010
Efavirenz	BCRP	P-GP, BCRP		OATP2B1	Storch et al., 2007; Weiss et al., 2007; Kis et al., 2010
Indinavir	P-GP			OATP1A2	Lee et al., 1998; Van Der Sandt et al., 2001; Campbell et al., 2015
Lopinavir	P-GP	P-GP, BCRP	OATP1A2		Lee et al., 1998; Janneh et al., 2007; Weiss et al., 2007; Hartkoorn et al., 2010
Nelfinavir	P-GP	P-GP, BCRP		OATP2B1	Kim et al., 1998; Gupta et al., 2004; Storch et al., 2007; Weiss et al., 2007; Kis et al., 2010
Nevirapine		P-GP, BCRP			Storch et al., 2007; Weiss et al., 2007
Raltregavir	P-GP, BCRP				Hashiguchi et al., 2013
Ritonavir	P-GP	P-GP, BCRP		OATP1A2, OATP2B1	Van Der Sandt et al., 2001; Gupta et al., 2004; Weiss et al., 2007; Hartkoorn et al., 2010
Saquinavir	P-GP	P-GP, BCRP	OATP1A2	OATP1A2, OATP2B1	Kim et al., 1998; Lee et al., 1998; Gupta et al., 2004; Janneh et al., 2005; Weiss et al., 2007; Hartkoorn et al., 2010; Kis et al., 2010
Zidovudine	P-GP, BCRP, MRP4	BCRP			Schuetz et al., 1999; Wang et al., 2003; Pan et al., 2007

Although the precise mechanisms, and effects, of alterations in BBB permeability associated with preeclampsia have not yet been elucidated, it is possible to gain an insight into the potential consequences of BBB modification from clinical situations in which the expression of BBB drug transporters and BBB integrity are altered. In this regard, ischemic and hemorrhagic stroke are life-threatening conditions whose outcomes include severe BBB disruption (Knowland et al., 2014; Keep et al., 2018). In rodent models of ischemic stroke, based on middle cerebral artery occlusion (MCAO), an increase in brain water content and a decrease in the expression of TJ proteins, including occludin and claudin-5, have been observed (Huang et al., 2017). In functional terms, the cited reports demonstrated an increase in BBB permeability, i.e., increased brain levels of drugs transported through the paracellular pathway. Interestingly, MCAO also resulted in a time dependent up-regulation of P-GP expression (Cen et al., 2013; DeMars et al., 2017), which may serve as a compensatory protective mechanism, especially for drugs that are substrates of this transporter.

**FIGURE 2:**
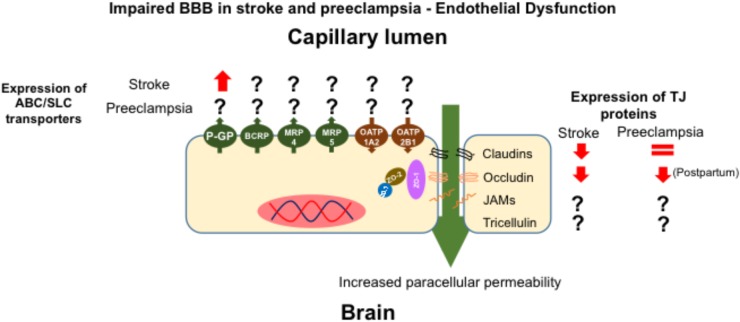
Effect of the endothelial dysfunction elicited by preeclampsia and stroke on the expression of transporters and TJ proteins. In both stroke and preeclampsia there is a decrease in the expression of TJ proteins, but in preeclampsia, this change appears to be evident post-partum. In stroke, endothelial dysfunction increases the expression of P-glycoprotein.

Pathological similarities are observed in both ischemic stroke and preeclampsia, including neuroinflammation, vasogenic edema and increased BBB permeability (Figure 2). To date, there is a lack of studies addressing the effect of preeclampsia on BBB physiology and, in particular, the effects of this disorder on TJ complexes, which govern paracellular permeability, and on ABC/SLC transporters, which regulate transcellular permeability. However, there is potential to monitor ABC transporter functionality, particularly P-GP activity, in women who have a history of preeclamptic pregnancies using non-invasive PET studies employing ^11^C-verapamil as tracer (Shin et al., 2016). Future studies could employ this technique in women who had suffered preeclampsia or eclampsia, in order to measure P-GP activity and investigate whether there is a correlation between transporter activity and propensity of seizures.

## Concluding Remarks

The BBB is a highly restrictive but dynamic system that regulates the transport of ions and molecules into and out of the brain. Consequently, alterations in its function could result in an increased CNS exposure to potentially toxic xenobiotics, including therapeutic drugs, and endogenous factors. Given the findings that preeclampsia is associated with increased BBB permeability, there is an urgent and fundamental need to characterize the functionality of BBB ABC and SLC transporter proteins involved in CNS drug disposition through the use of appropriate pre-clinical models and execution of clinical studies.

A better understanding of preeclampsia-associated changes in BBB physiology would not only allow characterization of the processes responsible for pathophysiological changes, but could help improve the therapeutic management of women experiencing, or those who had experienced, preeclamptic pregnancies. Indeed, this knowledge will help to reduce the risk of acute and chronic complications caused by alterations in BBB function elicited by preeclampsia.

## Author Contributions

PT-V designed and wrote the manuscript. CE and JP contributed to the writing of the manuscript and provided a critical revision of its contents.

## Conflict of Interest Statement

The authors declare that the research was conducted in the absence of any commercial or financial relationships that could be construed as a potential conflict of interest.
